# Therapeutic efficacy of artesunate-amodiaquine and artemether-lumefantrine combinations in the treatment of uncomplicated malaria in two ecological zones in Ghana

**DOI:** 10.1186/s12936-015-1080-x

**Published:** 2016-01-05

**Authors:** Benjamin Abuaku, Nancy Duah, Lydia Quaye, Neils Quashie, Keziah Malm, Constance Bart-Plange, Kwadwo Koram

**Affiliations:** Epidemiology Department, Noguchi Memorial Institute for Medical Research, College of Health Sciences, University of Ghana, P. O. Box LG581, Legon, Ghana; Centre for Tropical Clinical Pharmacology and Therapeutics, University of Ghana Medical School, P. O. Box GP4236, Accra, Ghana; National Malaria Control Programme, Public Health Division, Ghana Health Service, Accra, Ghana

**Keywords:** Therapeutic efficacy, Artesunate-amodiaquine, Artemether-lumefantrine, Uncomplicated malaria, Ecological zones, Ghana

## Abstract

**Background:**

Case management based on prompt diagnosis and adequate treatment using artemisinin-based combination therapy (ACT) remains the main focus of malaria control in Ghana. As part of routine surveillance on the therapeutic efficacy of ACT in Ghana, the efficacy of amodiaquine-artesunate (AS-AQ) and artemether-lumefantrine (AL) were studied in six sentinel sites representing the forest and savannah zones of the country.

**Methods:**

Three sites representing the two ecological zones studied AS-AQ whilst the other three sites studied AL. In each site, the study was a one-arm prospective evaluation of the clinical, parasitological, and haematological responses to directly observed therapy for uncomplicated malaria with either AS-AQ or AL among children aged 6 months and 9 years. The WHO 2009 protocol for monitoring anti-malarial drug efficacy was used for the study between July 2013 and March 2014.

**Results:**

Per-protocol analyses on day 28 showed an overall PCR-corrected cure rate of 100 % for AS-AQ and 97.6 % (95 % CI 93.1, 99.5) for AL: 97.2 % (95 % CI 92.0, 99.4) in the forest zone and 100 % in the savannah zone. Kaplan–Meier survival analysis showed similar outcomes. Prevalence of fever decreased by about 75 % after the first day of treatment with each ACT in the two ecological zones. No child studied was parasitaemic on day 3, and gametocytaemia was generally maintained at low levels (<5 %). Post-treatment mean haemoglobin concentrations significantly increased in the two ecological zones.

**Conclusions:**

Therapeutic efficacy of AS-AQ and AL remains over 90 % in the forest and savannah zones of Ghana. Additionally, post-treatment parasitaemia on day 3 is rare suggesting that artemisinin is still efficacious in Ghana.

## Background

Malaria remains a major public health problem in the world after decades of deployment of different interventions. Globally, an estimated 3.3 billion people are at risk of malaria infection and disease. It is estimated that in 2013 there were approximately 198 million cases of malaria worldwide, representing a decline of 30 % since year 2000. In the same year, malaria accounted for approximately 584,000 deaths worldwide, representing a decline of 47 % since year 2000. Africa remains the continent with the heaviest burden of malaria, accounting for about 90 % of all malaria deaths in the world. It is also estimated that about 78 % of all malaria deaths occur among children aged under 5 years [1]. In 2013, malaria was estimated to account for 44 % of all outpatient clinic visits and 22.3 % of all under-five deaths in Ghana [2].

Case management based on prompt diagnosis and adequate treatment remains a major focus of malaria control in Ghana. Other interventions include intermittent preventive treatment among pregnant women (IPTp), long-lasting insecticide-treated nets (LLINs), indoor residual spraying (IRS), and recently seasonal malaria chemoprevention (SMC) [3]. Evidence of the declining efficacy of chloroquine from 1998 to 2003, and the superiority of artemisnin-based combination therapy (ACT) resulted in the replacement of chloroquine with artesunate-amodiaquine (AS-AQ) combination in 2005 as first-line treatment for uncomplicated malaria in Ghana [4–6]. In 2008, artemether-lumefantrine (AL) and dihydroartemisinin-piperaquine (DHAP) were added as alternative first-line drugs for patients unable to tolerate AS-AQ [7].

Since 2005 the Noguchi Memorial Institute for Medical Research (NMIMR), in collaboration with the National Malaria Control Programme (NMCP), has been monitoring the therapeutic efficacy of first-line anti-malarial drugs from sentinel sites across Ghana with the view of providing continuous data to inform malaria treatment policy in the country. This paper covers the therapeutic efficacy of AS-AQ and AL across two ecological zones in Ghana during the period July 2013 to March 2014 using the 2009 WHO protocol for surveillance of anti-malarial drug efficacy [8].

## Methods

### Study design

The study was a one-arm prospective evaluation of the clinical, parasitological, and haematological responses to directly observed therapy for uncomplicated malaria with either AS-AQ or AL among children aged 6 months to 9 years in sentinel sites representing the savannah and forest zones of Ghana. The primary objective of the study was to assess cure rates in the two ecological zones whilst secondary objectives were to assess patterns of fever and parasite clearance as well as changes in haemoglobin levels and gametocyte carriage rates after treatment with AS-AQ and AL.

### Study sites

Therapeutic efficacy of AS-AQ was studied in the Yendi Municipal and Wa Regional Hospitals (representing the savannah zone) and Hohoe Municipal Hospital (representing the forest zone). Therapeutic efficacy of AL was studied in Navrongo War Memorial Hospital (representing the savannah zone) and Bekwai Municipal and Begoro District Hospitals (representing the forest zone).

The Yendi Municipal is located in the Northern region of Ghana with an estimated municipal population of 117,780 and an annual average rainfall of 1125 mm. Wa regional hospital is located within Wa municipal in the Upper West region of Ghana with an estimated municipal population of 107,214 and an annual average rainfall of between 840 mm and 1400 mm. The Navrongo War Memorial Hospital is located in the Kassena Nakana East district in the Upper East region of Ghana with an estimated district population of 109,944 and an annual average rainfall of 950 mm. Malaria transmission in the savannah zone of Ghana is generally perennial with marked seasonal variation and an annual entomological rate of about 418 infective bites per person per year [9–11, 13–15].

Hohoe is located in the Volta region of Ghana with an estimated municipal population of 262,046 and an annual average rainfall of 1300 mm. The rainfall pattern is bimodal with two distinct rainy seasons: April to July as major and September to November as minor. Malaria transmission in the municipality is intense and peaks with the two rainy seasons. Bekwai municipal is located in the Ashanti region of Ghana with an estimated municipal population of 118,024. Annual rainfall in the municipality is 1600–1800 mm with double maxima rainfall in May and October each year. Malaria transmission in the municipality is intense and perennial. Begoro is located in the Fanteakwa district in the Eastern region of Ghana. The Fanteakwa district has an estimated population of 108,614. Annual rainfall in the district is 1500–2000 mm with double maxima rainfall in June and October each year. Malaria transmission in the district is intense and perennial. Annual entomological inoculation rate in the forest zone can be as high as 866 infective bites per person per year [9, 10, 12, 16–18].

### Study population

Children aged between 6 months and 9 years with a history of fever suggestive of malaria were screened for the study. Blood samples from a finger prick were used to prepare thick and thin smears for malaria microscopy and haemoglobin level determination. Children who met the inclusion criteria were recruited into the study after parental consent had been obtained. Inclusion criteria included axillary temperature ≥37.5 °C or history of fever during the past 24 h; mono-infection with *Plasmodium falciparum*; parasite count ranging between 1000 and 250,000 per µl; haemoglobin level >5 g/dl; and absence of signs/symptoms of severe malaria.

### Treatment

All anti-malarials used in the study were fixed-dose combinations, and were supplied by the WHO, Geneva. For sites studying AS-AQ, children enrolled received single daily weight-based products from Sanofi Aventis. The products were 25/67.5 mg (batch number 1084, expiring 01/2015) for children weighing 4.5 to <9 kg; 50/135 mg (batch number 3109, expiring 02/2015) for children weighing 9 to <18 kg; and 100/270 mg (batch number 5433, expiring 01/2015) for children weighing 18 to <36 kg. For sites studying AL, children enrolled received weight-based 20/120 mg of Coartem^®^ (batch number F0785, expiring 05/2014) at 0, 8, 24, 36, 48, and 60 h. Children weighing 5 to <15 kg were given one tablet per hour of treatment; those weighing 15 to <25 kg were given two tablets per hour of treatment; and those weighing 25 to <35 kg were given three tablets per hour of treatment. All tablets were dissolved in water and given under direct observation by a study nurse and children were observed for 30 min to ascertain retention of anti-malarial. Children who vomited during the observation period were re-treated with the same dose of anti-malarial and observed for an additional 30 min. Children with repeated vomiting were given parenteral therapy with quinine as per national standard treatment guidelines and excluded from the study. All children were allowed use of antipyretics. Children who showed signs/symptoms of severe malaria were withdrawn from the study.

### Patient follow-up

Children enrolled into the study were followed-up for 28 days. Children were seen at the outpatient department on days 1, 2, 3, 7, 14, 21, and 28 (day of commencement of treatment was counted as day 0). At each follow-up visit, the children were clinically examined by a study Physician, who recorded findings on a Case Record Form (CRF). Parasitaemia levels (asexual and sexual) were assessed on days 2, 3, 7, 14, 21, 28, and any day within the 28-day follow-up period that a child is brought to the clinic with fever. Thick and thin smears were stained with 3 % Giemsa for 30–45 min for quantification of asexual parasites against 200 white blood cells using a hand tally counter. Sexual parasite counts were done per 1000 white blood cells. Parasitaemia levels were expressed per µl blood assuming white blood cell count of 8000 per µl blood. A smear was declared negative when examination of 100 thick-film fields did not show presence of asexual parasites. For quality control purposes, all blood slides were read by two qualified independent microscopists, and discordant readings were re-examined by a third qualified independent microscopist. Discordance was defined as differences between the first and second microscopists regarding presence/absence of asexual or sexual parasites; species diagnosis; and day 0 counts meeting the inclusion criterion of 1000–250,000 per µl blood. The first or second reading was taken as final depending on whichever agrees with the third reading. Filter paper blots were obtained on day 0 and at recurrence of parasitaemia for PCR genotyping. Merozoite surface proteins 1 and 2 (*msp1*, *msp2*), and glutamate-rich protein (*glurp*) were used to distinguish between re-infection and recrudescence [19]. Haemoglobin levels were assessed for all study children on days 0, 14, and 28 using an automated haematology analyzer (Sysmex KX-21N™).

### Data analysis

Per protocol analysis was used to assess treatment outcomes on day 28 for the two ecological zones based on the WHO 2009 criteria: Early treatment failure (ETF), Late Parasitological Failure (LPF), Late Clinical Failure (LCF), and Adequate Clinical and Parasitological Response (ACPR) [8]. Kaplan–Meier survival analysis was used to assess the cumulative incidence of treatment success over the 28-day follow-up period. Secondary outcomes were patterns of fever and parasite clearance assessed for the two ecological zones using proportions (with 95 % CI) of children febrile/with temperature ≥37.5 °C or parasitaemic within the first week of follow-up. Haematological responses were also assessed using pre- and post-treatment mean haemoglobin levels on days 0, 14 and 28. Proportions were compared using Chi square and Fisher’s exact tests. Normally distributed variables were compared using Student’s *t* test while skewed distributions such as parasite counts were log transformed before using the normal approximation. Differences were considered significant at *p* < 0.05.

### Ethics

The Institutional Review Board of the Noguchi Memorial Institute for Medical Research, University of Ghana, reviewed and approved the study. Written informed consent was obtained from each parent/guardian at the start of the study. Each parent/guardian was informed of the objectives, methods, anticipated benefits and potential hazards of the study. They were also informed that they were at liberty to withdraw their children from the study at any time without penalty.

## Results

### Baseline characteristics

A cumulative total of 310 children participated in the study in the two ecological zones: 140 received AS-AQ and 170 received AL. Of the children who received AS-AQ, 81 (57.9 %) were within the savannah zone. Of the children who received AL, 124 (72.9 %) were in the forest zone. For both anti-malarials, there were no significant differences between the two ecological zones, in terms of male/female ratio as well as baseline mean haemoglobin levels (Tables 1, 2). The majority of children who received AS-AQ were aged 5–9 years in the forest zone (55.9 %; 95 % CI 42.5, 68.6) and less than 5 years in the savannah zone (69.1 %; 95 % CI 57.8, 78.7). For the same group of children, mean axillary temperature was significantly higher in the savannah zone compared with the forest zone whilst geometric mean parasite count was significantly higher in the forest zone (Table 1). Among children who received AL, there were no significant differences in baseline characteristics between the two ecological zones. No child receiving AL was reported to have taken any anti-malarial within the 14-day period preceding visit to the clinic (Table 2). However, twelve children who received AS-AQ in the savannah zone were reported to have taken an anti-malarial within the same period preceding their visit to the clinic (14.8 %; 95 % CI 8.2, 24.8) (Table 1). Intake of anti-malarial drugs among the twelve children was reported to have been stopped between 1 and 11 days prior to their visit to the clinic; mean number of days being 6.0 (±3.6). None of the children who received AS-AQ in the two ecological zones was gametocytaemic at baseline. Among children who received AL, none in the savannah zone and 4 (3.3 %) in the forest zone were gametocytaemic at baseline.

**Table 1:** Demographic, clinical, parasitological, and haematological characteristics of patients treated with AS-AQ at baseline

Characteristic	Total (n = 140)	Ecological zone		p value
		Forest (n = 59)	Savannah (n = 81)	
Male/female	74/66	29/30	45/36	0.563
Age group				
<5 years	82 (58.6 %)	26 (44.1 %)	56 (69.1 %)	0.005
5–9 years	58 (41.4 %)	33 (55.9 %)	25 (30.9 %)	
Axillary temperature in °C				
Mean temperature (sd)^a^	37.8 (0.9)	37.5 (0.1)	38.1 (1.1)	0.000
Range (min, max)	35.2, 40.0	36.9, 37.5	35.2, 40.0	
Parasitaemia/µl				
Geometric mean	32,479	44,669	25,750	0.001
Range (min, max)	(1000, 228,040)	(1000, 228,040)	(1029, 170,840)	
Haemoglobin level in g/dl				
Mean (sd)^a^	8.7 (1.6)	8.7 (1.0)	8.7 (1.9)	1.000
Range (min, max)	(5.4, 13.9)	(6.0, 13.9)	(5.4, 13.9)	
Previous anti-malarial intake	12 (8.6 %)	0	12 (14.8 %)^b^	0.005

^a^Standard deviation

^b^Six children had taken AS-AQ and the other six had taken AL within the 14-day period preceding their visit to the clinic

**Table 2:** Demographic, clinical, parasitological, and haematological characteristics of patients treated with AL at baseline

Characteristic	Total (n = 170)	Ecological zone		p value
		Forest (n = 124)	Savannah (n = 46)	
Male/female	77/93	56/68	21/25	0.907
Age group				
<5 years	110 (64.7 %)	81 (65.3 %)	29 (63.0 %)	0.924
5–9 years	60 (35.3 %)	43 (34.7 %)	17 (37.0 %)	
Axillary temperature in °C				
Mean temperature (sd)^a^	38.1 (1.0)	38.2 (1.0)	37.9 (1.1)	0.093
Range (min, max)	35.6, 40.2	35.6, 40.2	35.9, 40.1	
Parasitaemia/µl				
Geometric mean	39,983	38,665	43,766	0.415
Range (min, max)	(1080, 240,840)	(1080, 239,890)	(1280, 240,840)	
Haemoglobin level in g/dl				
Mean (sd)^a^	10.2 (1.5)	10.2 (1.6)	10.3 (1.2)	0.695
Range (min, max)	(5.6, 14.4)	(5.6, 13.1)	(6.4, 14.4)	

^a^Standard deviation

### Primary outcomes

**Fig. 1:**
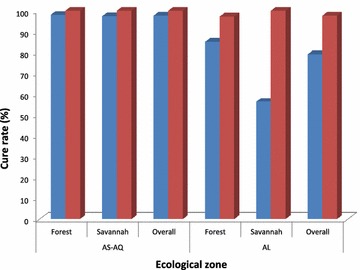
Per protocol PCR-uncorrected and PCR-corrected cure rates for AS-AQ and AL in two ecological zones in Ghana. *Colour blue* is PCR-uncorrected data whilst *dark red* is PCR-corrected data

Per protocol treatment outcomes on day 28 were assessed for a total of 278 evaluable children. Out of this, 125 received AS-AQ, and 153 received AL (Table 3). PCR-uncorrected analyses showed an overall day 28 cure rate of 97.6 % (95 % CI 93.1, 99.5) for AS-AQ: 98 % (95 % CI 89.4, 99.9) in the forest zone and 97.3 % (95 % CI 90.7, 99.7) in the savannah zone. The overall PCR-uncorrected cure rate for AL was 79.1 % (95 % CI 71.8, 85.1): 85 % (95 % CI 77.5, 90.9) in the forest zone and 56.3 % (95 % CI 37.7, 73.6) in the savannah zone. PCR-corrected analyses showed an overall cure rate of 100 % (95 % CI 96.2, 99.9) for AS-AQ and 97.6 % (95 % CI 93.1, 99.5) for AL. Within the forest zone, PCR-corrected cure rate was 100 % (95 % CI 90.9, 99.8) for AS-AQ and 97.2 % (95 % CI 92.0, 99.4) for AL. Within the savannah zone, PCR-corrected cure rates for AS-AQ and AL were both 100 % (Fig. 1). No early treatment failure (ETF) was observed following treatment with AS-AQ or AL.

**Table 3:** Per protocol day-28 treatment outcomes (PCR-uncorrected) for AS-AQ and AL by two ecological zones in Ghana

Anti-malarial drug	Treatment outcome	Total	Ecological zone	
			Forest	Savannah
AS-AQ		n = 140	n = 59	n = 81
	ETF	0	0	0
	LCF	1	1	0
	LPF	2	0	2
	ACPR	122	49	73
	NA^a^	15	9^b^	6^c^
AL		n = 170	n = 124	n = 46
	ETF	0	0	0
	LCF	8	8	0
	LPF	24	10	14
	ACPR	121	103	18
	NA^a^	17	3^d^	14^e^

^a^Not assessed

^b^One was withdrawn on day 2 because she was generally weak. Eight were lost to follow-up: four on day 7; two on day 14; and two on day 21

^c^All lost to follow-up: three on day 3; two on day 7; and one on day 14

^d^One was withdrawn on day 0 because of excessive vomiting after intake of medicine. One each was lost to follow-up on days 1 and 2

^e^All lost to follow-up: four on day 7; seven on day 14; and three on day 21

**Fig. 2:**
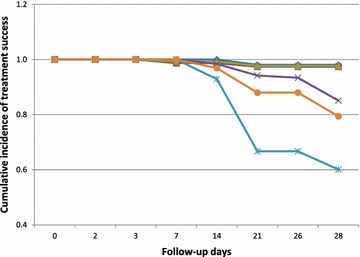
PCR-uncorrected Kaplan–Meier survival curve for AS-AQ and AL in two ecological zones in Ghana. *Dark blue line* with diamond spots represents AS-AQ in the forest zone; *dark red line* *with square spots* represents AS-AQ in the savannah zone; *green line with delta spots* represents combined or overall AS-AQ data (for both savannah and forest zones); *purple line with (X) spots* represents AL in the forest zone; *light blue line* with (*) spots represent AL in the savannah zone; and *orange line with circular spots* represents combined or overall AL data (for both savannah and forest zones)

PCR-uncorrected Kaplan–Meier survival analysis for AS-AQ showed cumulative treatment success incidence of 1.000 for the two ecological zones until day 7, when the incidence of success declined to 0.987 (95 % CI 0.917, 0.999) in the savannah zone and remained 1.000 in the forest zone (p = 0.895). Incidence of treatment success with AS-AQ remained 1.000 and 0.987 in the forest and savannah zones, respectively until day 21, when both zones experienced a decline to 0.981 (95 % CI 0.884, 0.999) and 0.974 (95 % CI 0.902, 0.996), respectively (p = 0.721), and remained same between day 21 and day 28. For AL, PCR-uncorrected treatment success remained 1.000 in the two ecological zones until day 14 when cumulative incidence of treatment success declined to 0.984 (95 % CI 0.936, 0.997) in the forest zone and 0.929 (95 % CI 0.795, 0.981) in the savannah zone (p = 0.208). Cumulative incidence of treatment success further declined on day 21 to 0.942 (95 % CI 0.880, 0.974) in the forest zone and 0.667 (95 % CI 0.481, 0.815) in the savannah zone (p < 0.001). Cumulative incidence of treatment success remained 0.942 in the forest zone until day 26, when it declined to 0.934 (95 % CI 0.870, 0.969), and further declined to 0.851 (95 % CI 0.772, 0.907) on day 28 compared with 0.601 (95 % CI 0.422, 0.766) on the same day in the savannah zone (p = 0.004) (Fig. 2). PCR-corrected Kaplan–Meier survival analyses showed a cumulative treatment success incidence of 0.979 (95 % CI 0.935, 0.993) for AL, translating into a cumulative treatment failure incidence of 0.021 (95 % CI 0.007, 0.065), and no treatment failure for AS-AQ.

### Secondary outcomes

**Fig. 3:**
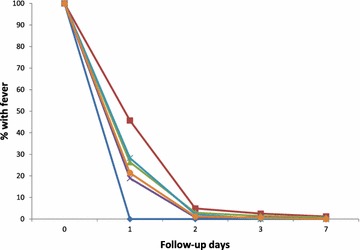
Proportion of children with fever during first week of follow-up by ecological zone. *Dark blue line with diamond spots* represents AS-AQ in the forest zone; *dark red line with square spots* represents AS-AQ in the savannah zone; *green line with delta spots* represents combined or overall AS-AQ data (for both savannah and forest zones); *purple line with (X) spots* represents AL in the forest zone; light blue line with (*) spots represent AL in the savannah zone; and *orange line with circular spots* represents combined or overall AL data (for both savannah and forest zones)

Generally, prevalence of fever decreased by about 75 % after first day of treatment with each ACT (26.4 %; 95 % CI 19.5, 34.7 for AS-AQ and 21.4 %; 95 % CI 15.6, 28.6 for AL; p = 0.372) (Fig. 3). For AS-AQ, only one child in the savannah zone was reported to have had fever on day 7 (1.2 %; 95 % CI 0.1, 8.0). This child was, however, aparasitaemic and remained same throughout the rest of the follow-up period. No child was febrile in the forest zone on day 7. For AL, no child was febrile on day 7 in the two ecological zones.

**Fig. 4:**
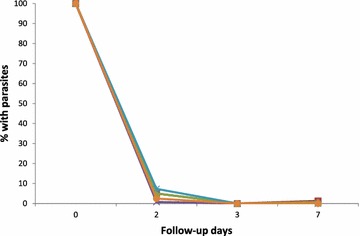
Proportion of children with parasites during first week of follow-up by ecological zone. *Dark blue line with diamond spots* represents AS-AQ in the forest zone; *dark red line with square spots* represents AS-AQ in the savannah zone; *green line with delta spots* represents combined or overall AS-AQ data (for both savannah and forest zones); *purple line with (X) spots* represents AL in the forest zone; light blue line with (*) spots represent AL in the savannah zone; and *orange line with circular spots* represents combined or overall AL data (for both savannah and forest zones)

No child in the two ecological zones was parasitaemic on day 3 (Fig. 4). For AS-AQ, only one child in the savannah zone was parasitaemic on day 7 (1.3 %; 95 % CI 0.1, 7.9) as against none in the forest zone. The child who had malaria parasites on day 7 was confirmed by PCR to be a case of re-infection. For AL, no child was parasitaemic on day 7.

Prevalence of gametocytaemia remained less than 5 % during the 28-day follow-up period after treatment with AS-AQ and AL in the two ecological zones. For AS-AQ, gametocytaemia was absent in the forest zone during the entire follow-up period. Gametocytaemia in the savannah zone was 1.3 % (95 % CI 0.1, 7.7) on days 2 and 3 after treatment with AS-AQ, and remained absent from day 7 to day 28. For AL, gametocytaemia in the forest zone declined from 3.3 % (95 % CI 1.1, 8.6) on day zero to 0.9 % (0.1, 5.5) on day 28 (p = 0.413), and was present only on day 7 (2.4 %; 95 % CI 0.1, 4.4) in the savannah zone.

Mean haemoglobin concentration in the forest zone significantly increased from 8.7 (±1.0) at baseline to 9.2 (±0.8) on day 28 after treatment with AS-AQ (p = 0.006). Similarly, mean haemoglobin concentration in the same zone significantly increased from 10.2 (±1.6) at baseline to 10.7 (±1.3) on day 28 after treatment with AL (p = 0.011). Within the savannah zone, mean haemoglobin concentration significantly increased from 8.7 (±1.9) at baseline to 10.3 (±2.1) after treatment with AS-AQ (p < 0.001) (Table 4).

**Table 4:** Changes in mean haemoglobin levels after treatment with AS-AQ and AL in two ecological zones in Ghana

Anti-malarial		Ecological zone			
		Forest	p value	Savannah	p value
AS-AQ	Day 0 mean Hb (sd)	8.7 (1.0)	0.295	8.7 (1.9)	
	Day 14 mean Hb (sd)	8.9 (1.0)	0.006	9.6 (1.8)	0.002
	Day 28 mean Hb (sd)	9.2 (0.8)		10.3 (2.1)	<0.001
AL	Day 0 mean Hb (sd)	10.2 (1.6)		10.3 (1.2)	
	Day 14 mean Hb (sd)	10.4 (1.3)	0.299	**	
	Day 28 mean Hb (sd)	10.7 (1.3)	0.011	**	

** Not available

## Discussion

The therapeutic efficacy of two of Ghana’s first-line anti-malarial drugs for the treatment of uncomplicated malaria (AS-AQ and AL) were studied in six sentinel sites representing the forest and savannah zones during the 2013/2014 surveillance period, with the view of providing information to guide malaria treatment policy in the country.

The study showed that PCR-uncorrected cure rates for AS-AQ were 98 % (95 % CI 89.4, 99.9) in the forest zone and 97.3 % (95 % CI 90.7, 99.7) in the savannah zone whilst AL showed PCR-uncorrected cure rates of 85 % (95 % CI 77.5, 90.9) in the forest zone and 56.3 % (95 % CI 37.7, 73.6) in the savannah zone. Although the study was not a comparative study, the findings compare well with the observation of higher PCR-uncorrected cure rate for AS-AQ, compared with AL, in Mozambique [20]. This observation may be explained by the longer terminal half-life of amodiaquine (approximately 10 days) over lumefantrine (approximately 4 days) resulting in a higher post-treatment prophylactic effect of AS-AQ [21, 22]. This notwithstanding, PCR-corrected cure rates for AS-AQ and AL were comparable in the forest and savannah zones of Ghana (100 % for AS-AQ and 97.2–100 % for AL) as in other studies [5, 22–26].

Both AS-AQ and AL achieved effective fever and parasite clearance in the two ecological zones. Prevalence of fever in both zones declined by 75 % after first day of treatment with each ACT; no child was parasitaemic on day 3; and prevalence of gametocytaemia generally declined from 3.3 to 0.9 % within the 28-day follow-up period. These findings suggest that ACT still has a rapid effect on fever and parasite clearance as well as gametocyte carriage in Ghana [5, 23, 27, 28]. Absence of parasitaemia on day 3 is an indication that Ghana is currently free of artemisinin resistance based on WHO’s indicator of day 3 parasitaemia of ≥10 % [29].

Post-treatment mean haemoglobin concentrations assessed on day 28, compared with pre-treatment concentrations, showed significant increase with both AS-AQ and AL treatment in the forest zone, and AS-AQ in the savannah zone (there was no available post-treatment data for AL treatment in the savannah zone). These findings suggest that ACT treatment for uncomplicated malaria has a positive effect on post-treatment haemoglobin levels. Though some studies have not shown significantly improved post-treatment haemoglobin levels after ACT treatment [26, 28, 30], others have [5, 27, 31].

## Conclusions

Therapeutic efficacy of AS-AQ and AL remains over 90 % in the forest and savannah zones of Ghana. The two drugs were effective in clearing fever and parasites as well as maintaining gametocytaemia at low rates (less than 5 %), and improving post-treatment mean haemoglobin levels. Artemisinin is still efficacious in Ghana by virtue of the absence of parasitaemia on day 3 post-treatment.
